# Typhoid Fever at Maidstone

**Published:** 1897-10-02

**Authors:** 


### TYPHOID FEVER AT MAIDSTONE.

The alarming outbreak of typhoid fever which is now running its course at Maidstone would appear from the accounts which we have received to be but another example of the want of care which is so often exhibited in all that pertains to the protection of the water supplies of our smaller towns. A consideration of the circumstances under which it has taken place raises questions which are of importance far away from the locality which is in this particular instance involved, and the first of these is as to how far it is ever wise to distribute unfiltered water.

If a large area of country can be obtained as a collecting ground, and can be kept for that purpose alone, then no doubt the springs arising from it form a safe water supply which may require no further purification. But the encroachments of towns, the incursion of town dwellers into country districts, and the extending use of town manure, all lead to the fouling of many sources of supply which used to be unexceptionable; and the opinion is rapidly gaining ground that the only choice lies between the rigid reservation of a collecting ground and the artificial production of a pure water by sand filtration.

The proof that sand filtration, if properly performed, is capable of providing a safe water is complete and apparently irrefutable, but the experience of every year tends more and more to shake the confidence which has so long been felt in springs and in deep wells.

Both the fine sand of the lower greensand formation, by which the town of Maidstone is surrounded, and the chalk of the South Downs, form admirable filters. But it has to be remembered that wherever there is a spring, and also wherever there is a deep well from which any quantity of water can be drawn, the water flows to the spring or to the well, not through the microscopic interstices in the sand or in the chalk, but through channels and through cracks, comparatively large spaces, and that these cracks and channels are quite as capable of being polluted as are leaky water-pipes, if a polluting substance be present in the neighbourhood.

The present outbreak at Maidstone shows how easily a spring may be polluted, just as the outbreak a few years ago at Worthing illustrated the danger of drawing from so reputedly safe a source as the chalk when in the neighbourhood of a town, and there can be little doubt that before very long the necessity of filtering all supplies except those drawn from a specially-reserved collecting ground will have to be seriously considered.

